# Position dependence of recovery coefficients in ^177^Lu-SPECT/CT reconstructions – phantom simulations and measurements

**DOI:** 10.1186/s40658-024-00662-y

**Published:** 2024-06-28

**Authors:** Julian Leube, Wies Claeys, Johan Gustafsson, Maikol Salas-Ramirez, Michael Lassmann, Michel Koole, Johannes Tran-Gia

**Affiliations:** 1 Department of Nuclear Medicine, University Hospital Würzburg, Oberdürrbacherstr. 6, Würzburg, 97080 Germany; 2 Department of Imaging and Pathology, KU Leuven, Herestraat 49, Leuven, 3000 Belgium; 3 Medical Radiation Physics Lund, Lund University, Skåne University Hospital Lund, Lund, 221 85 Sweden

**Keywords:** Quantitative SPECT/CT, SPECT, Harmonization, Recovery coefficients, Monte Carlo simulation, ^177^Lu SPECT/CT, Accreditation

## Abstract

**Background:**

Although the importance of quantitative SPECT has increased tremendously due to newly developed therapeutic radiopharmaceuticals, there are still no accreditation programs to harmonize SPECT imaging. Work is currently underway to develop an accreditation for quantitative ^177^Lu SPECT/CT. The aim of this study is to verify whether the positioning of the spheres within the phantom has an influence on the recovery and thus needs to be considered in SPECT harmonization. In addition, the effects of these recovery coefficients on a potential partial volume correction as well as absorbed-dose estimates are investigated.

**Methods:**

Using a low-dose CT of a SPECT/CT acquisition, a computerized version of the NEMA body phantom was created using a semi-automatic threshold-based method. Based on the mass-density map, the detector orbit, and the sphere centers, realistic SPECT acquisitions of all possible 720 sphere configurations of both the PET and the SPECT versions of the NEMA Body Phantom were generated using Monte Carlo simulations. SPECT reconstructions with different numbers of updates were performed without (CASToR) and with resolution modeling (STIR). Recovery coefficients were calculated for all permutations, reconstruction methods, and phantoms, and their dependence on the sphere positioning was investigated. Finally, the simulation-based findings were validated using SPECT/CT acquisitions of six different sphere configurations.

**Results:**

Our analysis shows that sphere positioning has a significant impact on the recovery for both of the reconstruction methods and the phantom type. Although resolution modeling resulted in significantly higher recovery, the relative variation in recovery within the 720 permutations was even larger. When examining the extreme values of the recovery, reconstructions without resolution modeling were influenced primarily by the sphere position, while with resolution modeling the volume of the two adjacent spheres had a larger influence. The SPECT measurements confirmed these observations, and the recovery curves showed good overall agreement with the simulated data.

**Conclusion:**

Our study shows that sphere positioning has a significant impact on the recovery obtained in NEMA sphere phantom measurements and should therefore be considered in a future SPECT accreditation. Furthermore, the single-measurement method normally performed for PVC should be reconsidered to account for the position dependency.

**Supplementary Information:**

The online version contains supplementary material available at 10.1186/s40658-024-00662-y.

## Background

With the fast development of new molecular radiotherapies (MRTs), quantitative ^177^Lu SPECT/CT imaging has gained considerable importance as the primary imaging tool for MRT dosimetry [1, 2]. Because of its increasing importance, there have recently been efforts to harmonize quantitative SPECT/CT imaging. Only through comparable MRT dosimetry across sites, principles for patient-specific MRT can be developed and applied clinically in the future. In addition, harmonization is crucial for the application of artificial intelligence in SPECT/CT imaging [3]. Due to the often small number of specific cases in nuclear medicine, it is possible to increase the amount of training data and thus decisively improve the efficiency of neural networks by data sharing across several centers. However, this is only possible through data harmonization, since machine learning methods are very sensitive to the distribution of the training data. Harmonization is also needed in the field of radiomics to make the analysis workflows more reliable and reproducible [4, 5].

The current effort to harmonize ^177^Lu SPECT/CT imaging is inspired by the European Association of Nuclear Medicine (EANM) Forschungs GmbH (EARL) accreditation for PET/CT [6, 7]. For this accreditation process, a PET/CT acquisition of a National Electrical Manufacturers Association (NEMA) International Electrotechnical Commission (IEC) PET Body Phantom (NU 2-2018) with sphere inserts (“NEMA PET Phantom”, sphere diameters: 10 mm, 13 mm, 17 mm, 22 mm, 28 mm, and 37 mm) is used to determine recovery coefficients (RCs) [8] for each of the six differently sized spheres. The RC is defined as PET-based sphere signal (activity or activity concentration) divided by the nominal (radionuclide calibrator-based) sphere signal. The ideal RC is a value close to 1, but lower values are typically observed in SPECT and PET imaging due to the partial volume effect (PVE). This refers to a quantitative bias caused by two effects: blurring of the activity distribution due to limited spatial resolution and sampling of the image into finite voxels, where the contours of the voxel may not match the distribution of the radiopharmaceutical [9, 10]. For the accreditation process, it is determined whether RCs are within a specified range of values for all spheres, which corresponds to a comparable resolution of the reconstructed images [11].

In recent years, several efforts have been made to harmonize SPECT imaging. A key foundation was provided by the MRTDosimetry project [12], in which eight sites across Europe achieved reproducible ^177^Lu SPECT/CT calibration factors and accurate activity quantification. By carefully harmonizing the setups in terms of system manufacturer, acquisition and reconstruction, harmonization of quantitative imaging performance between all sites was demonstrated based on a 3D-printed anthropomorphic phantom. However, the authors pointed out that the use of the NEMA PET Phantom for ^177^Lu SPECT harmonization is not ideal because the dimension of the smallest sphere is below the resolution limit of SPECT (1–2 cm for ^177^Lu and medium energy collimation). In addition, the volumes of many organs at risk, such as the kidneys and the spleen (volumes in the range of 100 mL to 250 mL [13]), are well beyond the largest sphere of the NEMA PET Phantom (volume of 26.5 mL). Therefore, the EANM working group, which is currently developing a protocol for a future ^177^Lu SPECT accreditation program [14], has proposed a modified phantom for SPECT harmonization. In what we will call “NEMA SPECT Phantom” (not to be confused with the SPECT Acceptance Testing Phantom as defined in NEMA NU 1-2018) throughout this manuscript, the smallest sphere from the NEMA PET Phantom (diameter of 10 mm) is replaced with a larger sphere of 60 mm diameter. This solves the issues related to resolution limits and the volume range covered. Another aspect that needs to be investigated for the accreditation program is the influence of the sphere arrangement. Since the resolution of a PET/CT system is spatially invariant in good approximation, only a small effect of the sphere positioning on RC is expected for PET [15]. Recently Gabiña et al. [16] derived a formula to calculate the theoretical RC curve for a SPECT system in dependence of the resolution. They assume a linear translation invariant system, which is a very simple model of a SPECT image. In real SPECT imaging, however, the modeling of a point spread function to achieve spatially invariant resolution is challenging and convergence is much slower. The former is due to the combination of collimation and the non-circular orbit of the gamma camera. During the measurement, the detector minimizes the distance to the surface of the phantom to ensure the best possible resolution for each acquired projection [17]. Furthermore, the most commonly used reconstruction methods in molecular imaging today, i.e., the maximum-likelihood expectation maximization and ordered subset expectation maximization (OSEM) algorithms, are known to be non-linear, leading to the effective spatial resolution being dependent on both the object considered and the environment (activity distribution in the entire field of view of the camera) in which the object is situated [18]. This could indeed affect the spatial resolution in the reconstructed image and result in a position dependence of the RC values. Furthermore, a direct consequence of the non-linearity of OSEM reconstruction is that the arrangement of the spheres might have an impact, because the reconstruction algorithm needs to correctly separate the counts from different spheres along each projection line. Additionally, the configuration of the spheres could potentially result in small distances between the surfaces of two adjacent spheres. Due to the limited SPECT resolution, there is spill-out of activity, which could potentially cause spill-in into the other sphere; resulting in a deviation in the RCs.

A potential influence of the sphere positioning on the RC values could also have an impact on MRT dosimetry, as partial volume corrections (PVCs) based on RCs are often applied here [19]. This approach will be referred to below as “RC-based PVC”: First, a NEMA PET/SPECT phantom with known activity concentration is scanned using the clinical acquisition protocol. Then, a curve is fitted to the calculated RCs of the spheres of the NEMA PET Phantom as function of the sphere diameter. Then, the diameter of the spherical lesion to be corrected is determined – preferably based on morphological imaging such as CT. Next, the activity within the lesion is determined using a SPECT or PET volume of interest (VOI) of the same volume. Finally, the activity is divided by the theoretical RC of a sphere of the size of the lesion, determined using the fitted recovery curve. Therefore, any dependence of RC on the sphere positioning will also affect the subsequent absorbed-dose estimates. For this correction, it is necessary that the lesion is also approximately spherical. Two recent studies have shown that the shape of the lesion can also be taken into account in the correction by determining the surface area to volume ratio [20, 21].

In this study, we investigate the impact of sphere positioning in the NEMA SPECT Phantom on RCs determined by ^177^Lu SPECT/CT. For this purpose, SPECT acquisitions for all possible sphere configurations (6! = 720 permutations) were generated using the Monte Carlo radiation transport simulation program SIMIND [22]. Subsequently, SPECT reconstructions were performed with and without resolution modeling (resolution recovery, RR), and a set of RCs was determined for each permutation. Based on these data, the variation of RC as a function of sphere positioning was determined for each sphere and for both reconstructions. Finally, the results were experimentally validated by ^177^Lu SPECT/CT measurements of six different sphere configurations of a NEMA SPECT Phantom.

## Methods

### SPECT/CT measurement of NEMA SPECT phantom to set up monte carlo simulations

A SPECT/CT measurement with a cold NEMA SPECT Phantom (i.e., the background compartment was filled with non-radioactive water) was performed at the University Hospital Würzburg to extract the most important phantom features for generation of a computerized NEMA Phantom. The measurements were performed with a Siemens Intevo Bold SPECT/CT system (9.5 mm crystal, medium-energy low penetration collimator). SPECT was performed to measure a realistic detector orbit (180° detector configuration, automatic contouring, continuous mode, 2 × 60 views). A high resolution CT (*CT-HD*, CTDI_Vol_ = 3.12 mGy, 28 mAs, 130 kVp, pitch factor 1.5, slice thickness 1 mm) was performed to determine the sphere positions inside the phantom. In addition, a standard CT (*CT-AC*, CTDI_Vol_ = 2.90 mGy, 28 mAs, 130 kVp, pitch factor 1.5, slice thickness 3 mm) was performed to generate a realistic mass-density map. Both CT images had an in-plane pixel size of 0.98 × 0.98 mm^2^ with a matrix size of 512 × 512 pixels. Based on the *CT-HD*, the sphere centers were determined using a semi-automatic thresholding method, the details of which are explained in the Supplementary Material.

### Monte carlo simulations for all possible sphere permutations

**Fig. 1:**
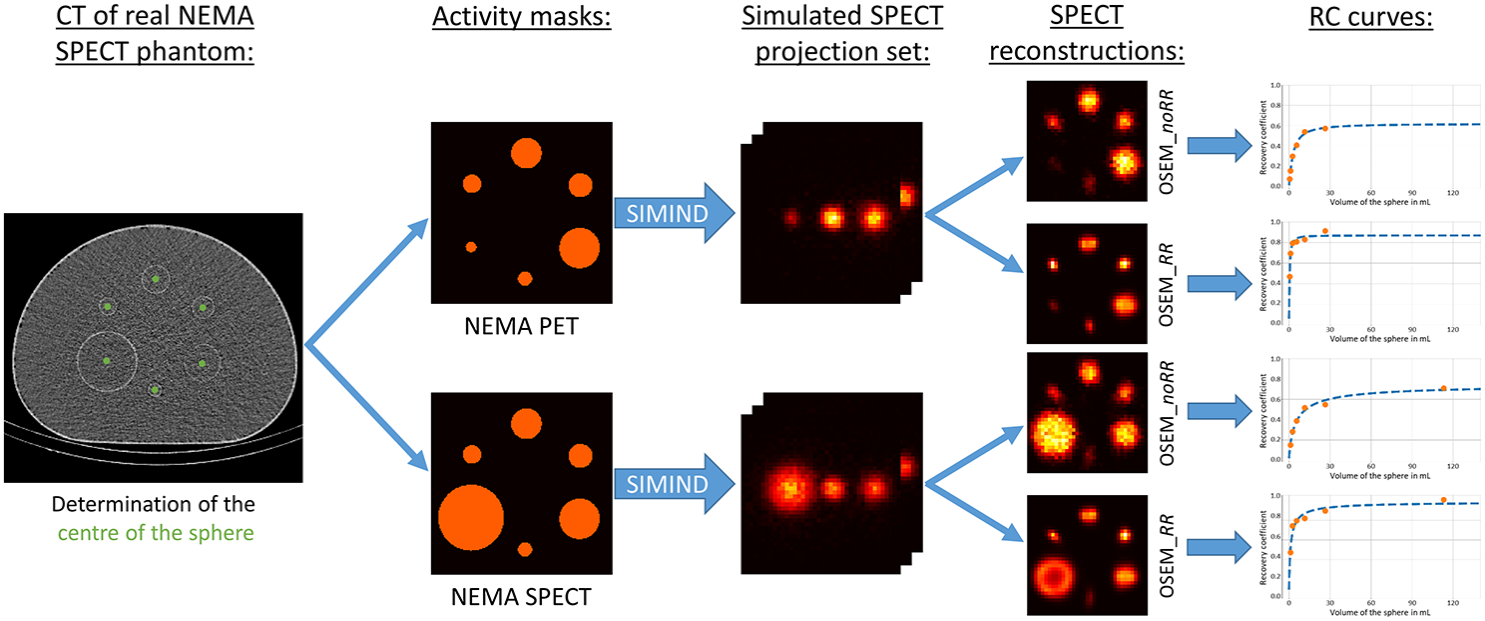
Schematic overview of the generation of the dataset utilized in this study

**Fig. 2:**
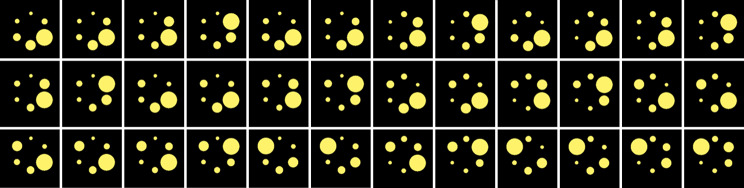
Example permutations of the computerized NEMA SPECT Phantom

Monte Carlo (MC) simulations were performed in SIMIND [22] to determine the influence of sphere positioning on the RCs. Figure 1 gives a schematic overview of the workflow used to generate the dataset used in this study. First, *CT-AC* was interpolated to a voxel size of 2.4 × 2.4 × 2.4 mm^3^ (matrix size of 256 × 256 × 256). Next, the HU values of the CT were converted to mass density by using a pre-determined, scanner-specific two-segment linear function [23]. The mass density map was then used as input for SIMIND to correctly simulate photon attenuation. The distance between detector and image center was extracted from the DICOM header and used for all performed simulations. The simulations were performed using SIMIND’s *multiple sphere routine*, where sphere centers ${R}_{j}$ and diameters ${d}_{j}$ are given as an input. The sphere diameters were permuted to create all 720 possible sphere configurations at the six designated positions, resulting in 720 simulations. In theory, mirroring the phantom in the sagittal plane along the gantry axis would represent an equivalent spherical arrangement, which would only require 360 simulations. However, the trajectory is not axisymmetric due to a rail system below the bed, which is why all 720 sphere permutations were performed. Examples of permutations of the computerized NEMA SPECT Phantom are given in Fig. 2. Strictly speaking, a separate mass density map would have to be created for each combination. However, the same mass density map can be used for all permutations without meaningful inaccuracies in the simulated attenuation, as the walls of the spheres are thin (around 1 mm according to manufacturer specification), and the attenuation coefficient is similar to that of the surrounding water (60 HU Vs.0 HU).

The simulation was set to recreate a measurement on the SPECT/CT system described above (Siemens Intevo Bold) using a 20% main energy window at 208 keV and two adjacent 10% scatter windows. In addition to the NEMA SPECT Phantom, simulations of the NEMA PET Phantom were also performed by replacing the 60 mm sphere by the 10 mm sphere. This resulted in a total of $2\times 720=\text{1,440}$ simulations, which were performed on the local high-performance computing cluster at the University of Würzburg. The variance-reduction in the SIMIND program makes the noise from the Monte Carlo simulations not following a Poisson distribution. Hence, a large number of histories were run in order to achieve convergence in simulated projections. After scaling the projections (activity concentration 2 MBq/mL), Poisson noise was added to generate realistic projections.

### SPECT reconstructions of simulations

In order to perform reconstructions for such a large number of simulations in a time-efficient manner, open-source reconstruction programs that provide batch reconstruction capabilities were used. Ordered Subset Expectation Maximization (OSEM) reconstructions without RR (OSEM_*noRR*) were performed using CASToR [24] (attenuation correction (AC), scatter correction (SC) with triple energy window (TEW) method, voxel size: 4.8 × 4.8 × 4.8 mm^3^, matrix size: 128 × 128 × 128) with 10 subsets and an increasing number of iterations (1 to 10 with step size 1, corresponding to 1 to 100 updates). Additionally, OSEM reconstructions with RR (OSEM_*RR*) were performed using STIR [25] (AC, SC with TEW, voxel size: 4.8 × 4.8 × 4.8 mm^3^, matrix size: 128 × 128 × 128) with 10 subsets and an increasing number of iterations (2 to 20 with step size 2, corresponding to 2 to 200 updates), a reconstruction very similar to the Siemens Flash3D reconstruction. Both reconstructions were performed for all $2\times 720=\text{1,440}$ permutations of the NEMA PET Phantom and the NEMA SPECT phantom, respectively.

### Determination of recovery coefficients of the permutations

To determine a set of six RC values for each configuration, both SPECT reconstructions (OSEM_*noRR* and OSEM_*RR*) were interpolated to *CT-HD* resolution using tri-linear interpolation. Although linear interpolation can lead to a small degradation in recovery, it was applied here in order to compare recovery using the high-resolution activity mask. An alternative approach would have been to interpolate the segmentation mask to SPECT resolution. However, this would have resulted in a more pronounced sampling effect due to the larger SPECT voxels. Then, a set of RC values was calculated for both phantoms, both reconstruction types, and all numbers of iterations (10 different for OSEM_*noRR and* OSEM_*RR*), as described in the Supplement. The RC is defined as the SPECT-based total activity in the sphere divided by the nominal activity within the sphere. For each sphere the mean RC ($\overline{RC}$), the maximum RC (${RC}_{max}$) and the minimum RC (${RC}_{min}$) over all 720 permutations were determined.

To quantify a potential spread of RC for each sphere, the RC variation ${\vartheta }_{RC}$ was calculated as the difference between ${RC}_{max}$ and ${RC}_{min}$ divided by $\overline{RC}$ (Table 1):

**Table 1:** Statistical analysis of the RCs for all 720 permutations

Phantom type	Reconstruction	Sphere diameter $d$	Mean $\overline{RC}$	RC variation ${\vartheta }_{RC}$
NEMA PET	OSEM_*noRR*	10	0.090	96%
		13	0.161	61%
		17	0.267	40%
		22	0.388	27%
		28	0.501	19%
		37	0.596	15%
	OSEM_*RR*	10	0.410	60%
		13	0.642	44%
		17	0.787	27%
		22	0.846	22%
		28	0.879	16%
		37	0.892	11%
NEMA SPECT	OSEM_*noRR*	13	0.098	110%
		17	0.183	56%
		22	0.292	32%
		28	0.406	21%
		37	0.517	15%
		60	0.718	10%
	OSEM_*RR*	13	0.566	48%
		17	0.751	27%
		22	0.829	21%
		28	0.868	15%
		37	0.880	11%
		60	0.997	7%

The mean RC ($\overline{RC}$) and the RC variation, ${\vartheta }_{RC}$ (see Eq. 1) of all 720 permutations are given for the two different phantom types (NEMA PET Phantom, NEMA SPECT Phantom) and both reconstructions (OSEM_*noRR*, OSEM_*RR*)

1 $${\vartheta }_{RC}=\frac{{RC}_{max}-{RC}_{min}}{\overline{RC}}.$$

### Fit of the RC curve

For each of the 1,440 simulations, a non-linear least squares fit of the RC curve (RC values plotted against the sphere diameter) was performed for both SPECT reconstructions (OSEM_*noRR*, OSEM_*RR*) using the *curve_fit* function ( [26], non-linear least squares fit) of the Python package *scipy*, with the following fit function [27, 28]:

2 $${f}_{RC}\left(d\right)={\left(1+{\left(\frac{\beta }{d}\right)}^{\gamma }\right)}^{-1}$$

For each fit, the values of $\beta$ and $\gamma$, as well as the coefficient of determination ${r}^{2}$ were determined. A mean fit $\overline{{f}_{RC}}\left(d\right)$ was then determined over all 720 permutations of each phantom type as follows:

3 $$\overline{{f}_{RC}}\left(d\right)=\frac{1}{720}\sum _{i=1}^{720}{f}_{{RC}_{i}}\left(d\right)$$

The standard deviation ${\sigma }_{RC}\left(d\right)$ was calculated using the following formula:

4 $${\sigma }_{RC}\left(d\right)=\sqrt{\frac{1}{719}{\sum }_{i=1}^{720}{\left({f}_{{RC}_{i}}\left(d\right)-\overline{{f}_{RC}}\left(d\right)\right)}^{2}}$$

Just as for RC (see Eq. 1), the variation of $\beta$ or $\gamma$ over all permutations is determined by the following formulas:

5 $${\vartheta }_{\beta }=\frac{{\beta }_{max}-{\beta }_{min}}{\stackrel{-}{\beta }}\,\,\, \text{and}\, \,\,{\vartheta }_{\gamma }=\frac{{\gamma }_{max}-{\gamma }_{min}}{\stackrel{-}{\gamma }}$$

In order to determine the global goodness of the fits to the determined RC values, the coefficient of determination ${r}^{2}$ was calculated for each fit performed.

### SPECT measurements for different sphere positioning

**Fig. 3:**
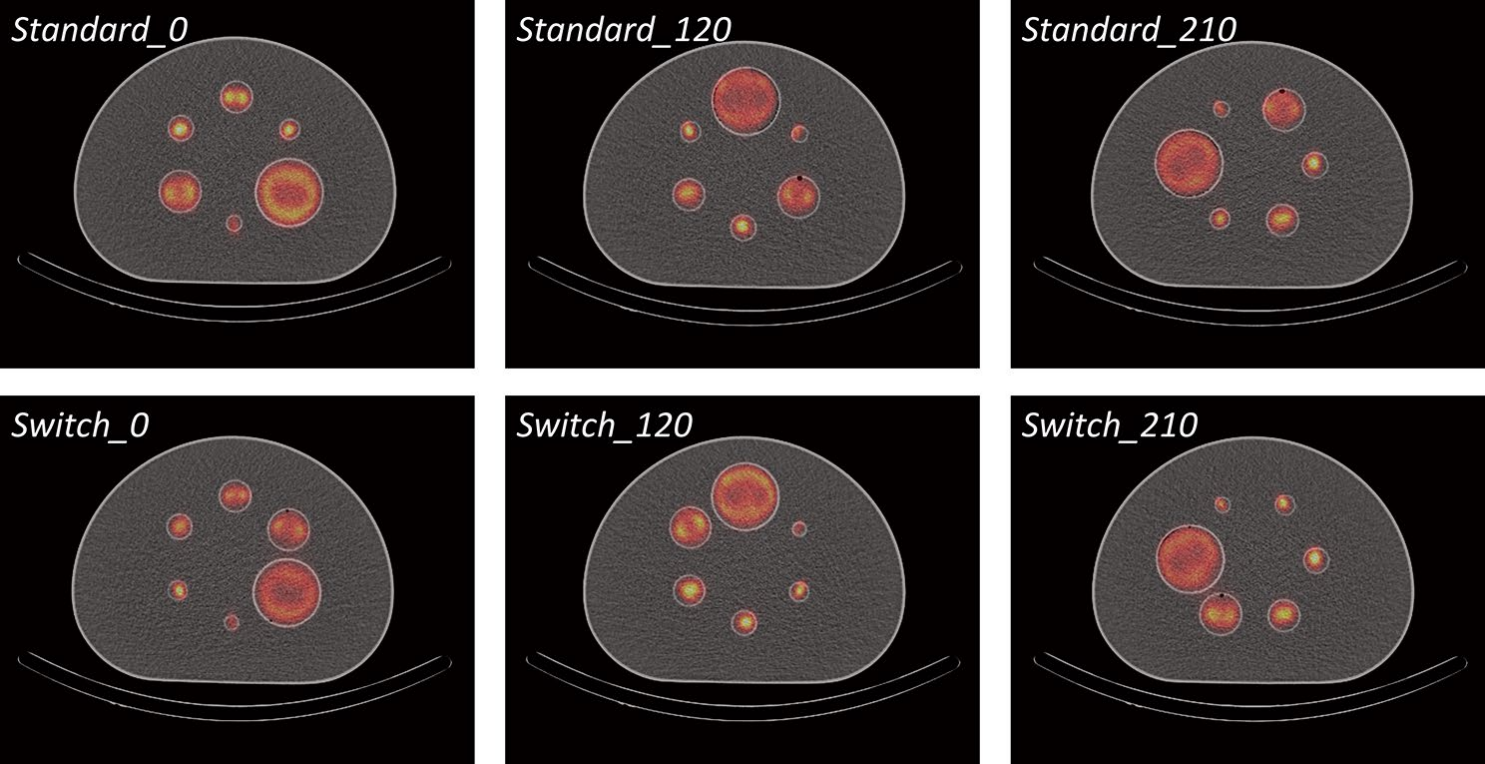
Overview of the six different sphere configurations used for the SPECT/CT measurements at the University Hospitals Leuven. The figure depicts the axial slice of the SPECT/CT fusion based on *CT-HD* and Flash3D SPECT reconstruction for all measured sphere configurations. Top from left to right: *Standard_0*, *Standard_120*, and *Standard_210*. Bottom from left to right: *Switch_0*, *Switch_120*, and *Switch_210*

In order to test the simulation-based observations under real measurement conditions, additional ^177^Lu SPECT/CT measurements were performed on a NEMA SPECT Phantom (different copy of the same phantom) at the University Hospitals Leuven. These measurements were based on the preliminary accreditation protocol given by EARL. In total, six different sphere configurations were measured. A *Standard* set of sphere configurations, where the spheres were positioned with alternating sphere sizes (large sphere surrounded by the two smallest spheres; this positioning is proposed in the preliminary EARL accreditation protocol (cite)) and then the entire sphere insert was rotated by 0°, 120°, and 210° (*Standard_0*, *Standard_120, Standard_210*); and a second set of sphere configurations (*Switch*) in which the smallest to the largest sphere were arranged clockwise and again rotating the entire sphere insert by 0°, 120° and 210° (*Switch_0, Switch_120, Switch_210*). An overview of all six sphere arrangements is given in Fig. 3. The measurements were performed with the same camera model as the one reproduced in the simulations (Siemens Intevo Bold with 9.5-mm crystal thickness and medium-energy low-penetration collimator) using the same imaging (180° detector configuration, 60 projections for each detector, time per projection of 20 s (this resulted in the 25 kcts per detector and projection proposed in the preliminary EARL protocol), matrix size: 256 × 256, pixel size: 2.4 × 2.4 mm^2^, a 20% main energy window at 208 keV and two adjacent 10% scatter windows) and reconstruction parameters (Flash3D with RR, AC, SC with TEW, voxel size: 2.4 × 2.4 × 2.4 mm^3^, matrix size: 256 × 256 × 128, 2 subsets, and an increasing number of 5, 10, 15, 20, 25, 30, 40 and 80 iterations). From a batch of no carrier added lutetium trichloride (^177^LuCl_3_, EndolucinBeta, Isotope Technologies Munich), a syringe containing a target activity of 400 MBq was prepared using a well-type activity meter (–model VIK-202 with IBC-LITE software, Comecer). This activity was used to prepare a 200 mL stock solution from which the spheres were filled, resulting in a uniform activity concentration of 2 MBq/mL. To prevent the lutetium from sticking to the sphere walls, approximately 1 g of ethylenediaminetetraacetic acid was added to the stock solution. A high-resolution CT (*CT-HD*, CTDI_Vol_ = 10.20 mGy, 150 mAs, 110 kVp, pitch factor of 0.8, axial resolution of 1 mm) was acquired to determine the sphere positions, and a low-resolution CT (*CT-AC*, CTDI_Vol_ = 10.20 mGy, 150 mAs, 110 kVp, pitch factor of 0.8, slice thickness of 5 mm) was acquired for attenuation correction. Both CT images had an in-plane resolution of 0.98 × 0.98 mm^2^ with a matrix size of 512 × 512 pixels. The segmentation masks used to calculate the RCs were obtained using the same methodology as applied for the creation of the activity masks for the simulations (NEMA SPECT Phantom measurement at the University Hospital Würzburg). The RCs for all six sphere configurations were calculated for all different numbers of Flash3D iterations using Eq. 3 of the Supplementary Material. In addition, a fit of the RC curve was performed for each of the six measurements using the fit function given by Eq. 2.

### Comparison between measurements and simulations

The agreement between the effects of sphere positioning as predicted by MC simulations and physical-phantom measurements was investigated. For this purpose, the RC curves of the six measured sphere configurations and of the simulations with the same sphere configuration were calculated. Due to an additional 30° rotation of the entire sphere insert against the sphere positions used for the simulations, the sphere configurations *Standard_210* and *Switch_210* were not present in the 720 simulated sphere permutations of our analysis. Therefore, additional SIMIND simulations were performed for these two configurations using the same parameters as the previous simulations. Given the visual similarity of the simulated and measured curves, it can be reasonably assumed that predictions about the specific RCs for each sphere configuration can be made based on the simulations. Additionally, two metrics were calculated to assess the deviation between measurements and simulations quantitatively:

6 $${\updelta }\left(\text{d}\right)= \frac{1}{6}{\sum }_{j=1}^{6}\left|R{C}_{measured,j}\left(d\right)-R{C}_{simulated,j}\left(d\right)\right|$$

7 $${\Delta }\left(\text{d}\right)= \frac{1}{6}{\sum }_{j=1}^{6}\left|R{C}_{measured,j}\left(d\right)-{\overline{RC}}_{measured}\left(d\right)\right|,$$

where $j$ is one of the six measured configurations, $R{C}_{measured,j}\left(d\right)$ is the RC curve of the measurements and $R{C}_{simulated,j}\left(d\right)$ is the RC curve of the simulations of these configurations. ${\overline{RC}}_{measured}\left(d\right)$ is the mean RC curve over all six measured configurations.

## Results

### Influence of the number of OSEM updates on the recovery coefficients

**Fig. 4:**
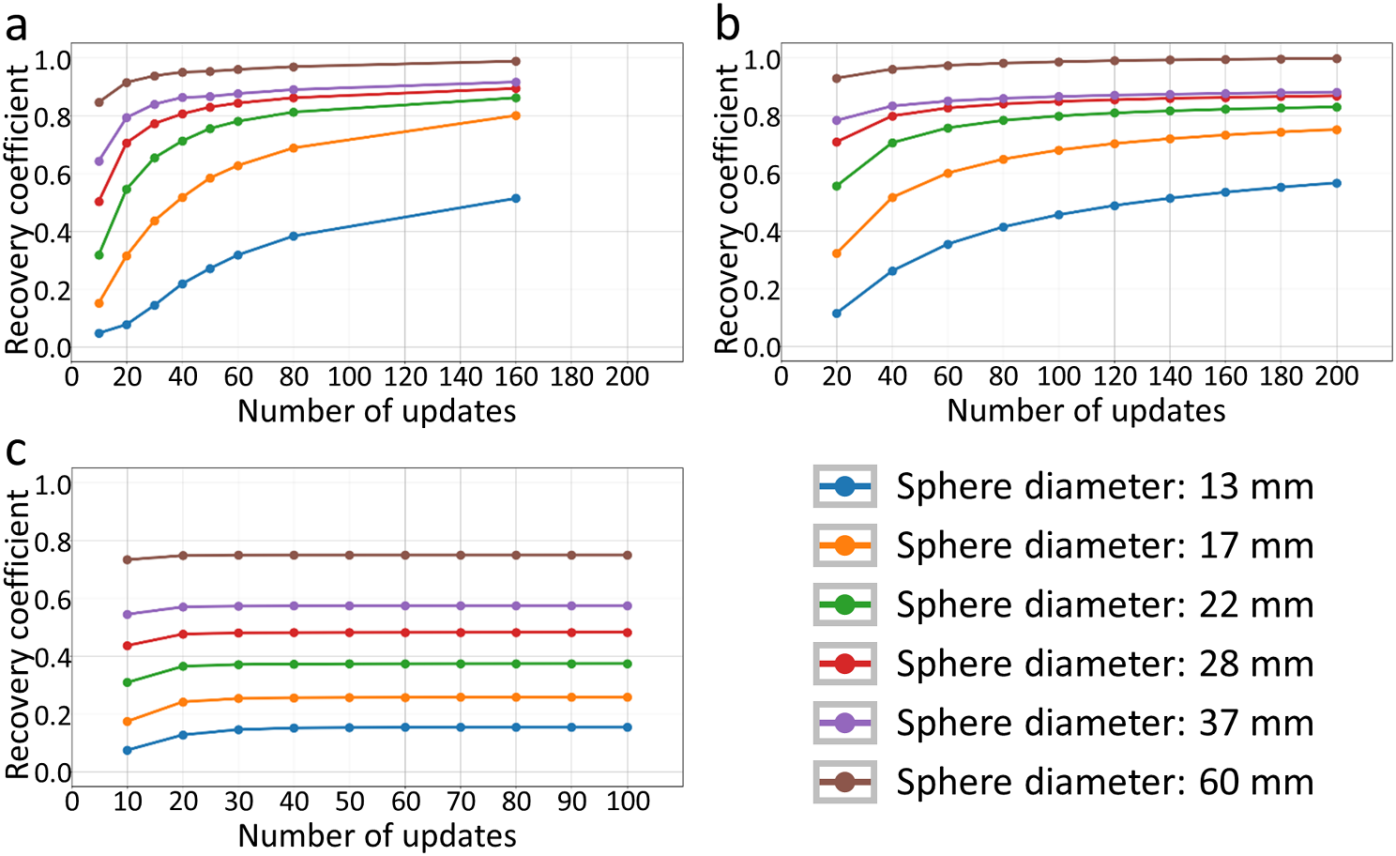
Recovery coefficient (RC) for the NEMA SPECT Phantom as a function of the number of updates. For each subfigure (**a**-**c**) the mean RC over all permutations for each sphere (different colors) is plotted as a function of the number of updates (number of iterations × number of subsets). **a**: Mean RC of SPECT/CT measurements of six sphere configurations using Flash3D reconstruction. **b**: RC averaged over all 720 simulated permutations using OSEM_*RR* reconstruction. **c**: RC averaged over all 720 simulated permutations using OSEM_*noRR* reconstruction

Figure 4 shows the RCs of the NEMA SPECT Phantom averaged over all 720 permutations as a function of the number of iterations. In addition, the RCs averaged over the six measured sphere configurations (see Fig. 3) are shown as function of the number of iterations. For OSEM_*noRR*, the recovery coefficient remains almost unchanged after 50 updates (Fig. 4a). This indicates that the activity estimates from images reconstructed without RR converge at an update number of about 50. Therefore, only OSEM_*noRR* reconstructions with 10 subsets and 5 iterations will be used in the further analysis. For OSEM_*RR*, a different convergence behavior can be observed for different spheres. While the largest sphere seems to be completely converged after just under 100 updates, the smallest sphere has not yet reached convergence even after 200 updates (Fig. 4b). The same behavior can be seen in the SPECT/CT measurements (Fig. 4c).

For further analysis, the reconstructions with the highest observed RCs were used, which corresponds to the highest number of updates performed in this study (OSEM_*RR*: 10 subsets with 20 iterations, or 200 updates; Flash3D: 2 subsets with 80 iterations, or 160 updates). Reconstructions with more updates were not performed in this study because of high computational cost and the increase in noise with the number of updates.

### Variation of recovery coefficient for different sphere positioning

**Fig. 5:**
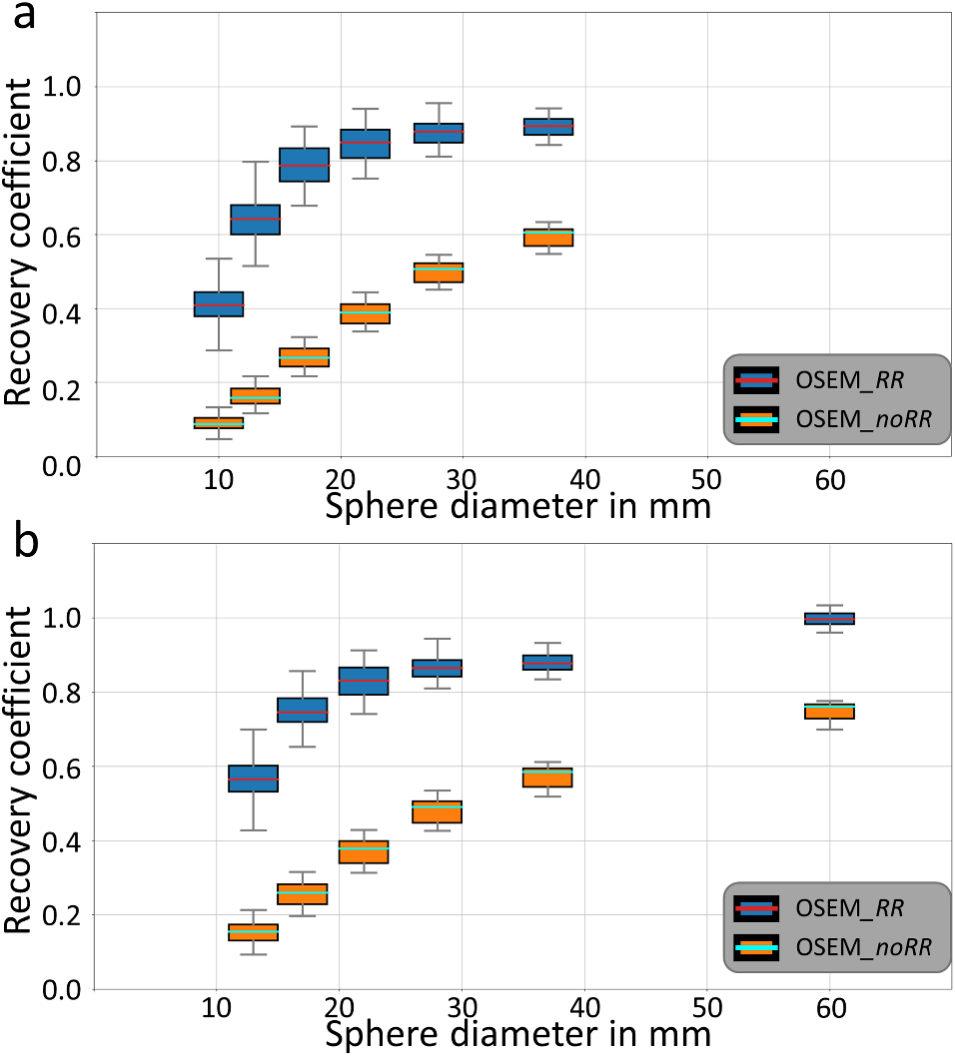
Boxplots showing the RCs of all permutations. Boxplots are separated by diameter (horizontal axis), reconstruction method (blue: OSEM_*RR, orange*: OSEM_*noRR*), and phantom type (**a**: NEMA PET Phantom, **b**: NEMA SPECT Phantom). The red (OSEM_*RR*) and cyan (OSEM_*noRR*) lines represent the median RC of all 720 permutations. The blue (OSEM_*RR*) and orange (OSEM_*noRR*) boxes represent the interquartile range of the RCs. The whiskers correspond to the maximum/minimum values for each sphere diameter

Figure 5 shows boxplots of RCs for all permutations separated by diameter, reconstruction method, and phantom type. Table 1 lists the corresponding mean values. The standard deviations and minimum/maximum values are given in Supplementary Table 1. For both phantoms, OSEM_*noRR* resulted in lower RCs than OSEM_*RR* (blue versus orange boxes in Fig. 5). The ratio between the mean RC of OSEM_*RR* and OSEM_*noRR* is very large for the smallest sphere (4.6 for NEMA PET and 5.8 for NEMA SPECT phantom) and decreases with increasing sphere size (1.5 and 1.4, respectively, for the largest sphere). The greatest improvement in RC through resolution recovery is achieved for the smallest sphere of the NEMA SPECT phantom, and the smallest is achieved for its largest sphere. Furthermore, a strong dependence of the sphere positioning on the RC was found for both phantoms, both reconstruction methods and all spheres (boxplots in Fig. 5). It can be observed that ${\vartheta }_{RC}$ (see Eq. 1) decreases with increasing sphere diameter, and that ${\vartheta }_{RC}$ is smaller for OSEM_*RR* than for OSEM_*noRR* reconstructions. In addition, the mean RC for spheres present in both phantoms (13 mm, 17 mm, 22 mm, 28 mm, and 37 mm) is smaller in the NEMA SPECT Phantom than in the NEMA PET Phantom.

### Investigating the sphere positioning resulting in minimal/maximal recovery coefficient for each sphere

**Fig. 6:**
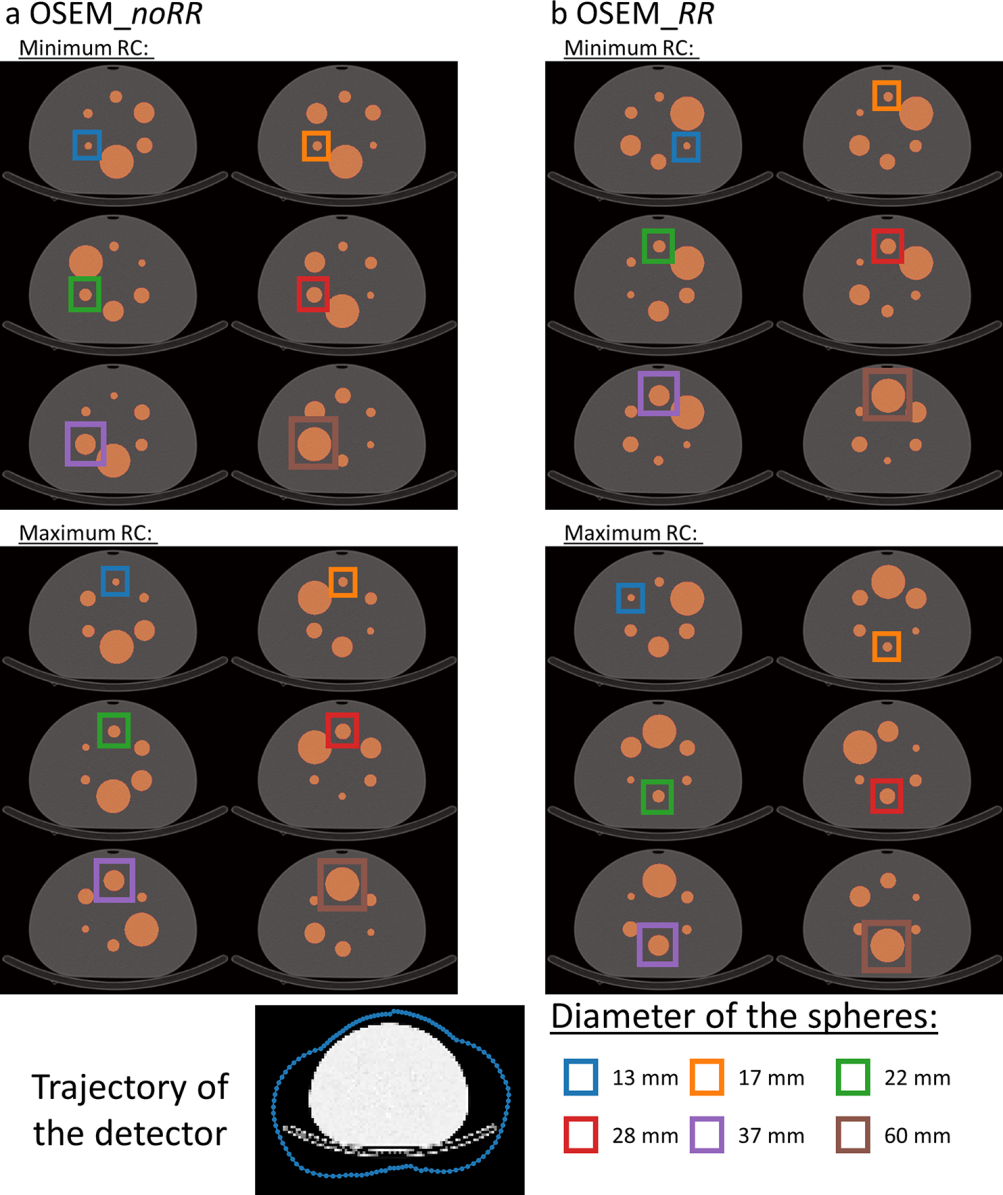
Permutations of the NEMA SPECT Phantom with the lowest/highest RCs. The plot shows the permutations of the NEMA SPECT Phantom with the lowest (top) and highest (bottom) RCs for reconstructions without (**a**: OSEM_*noRR*) and with resolution modeling (**b**: OSEM_*RR*). The positions of the sphere achieving the highest/lowest RC are depicted by colored boxes. The bottom left shows the detector orbit used in the simulations and the numbering of the positions, while the bottom right explains the sphere colors

In order to better understand the variation of RC shown in Fig. 5, the permutations resulting in the largest and smallest RC for each sphere were visually investigated. Figure 6 illustrates the permutations resulting in${RC}_{max}$ or ${RC}_{min}$ for the NEMA SPECT Phantom and both reconstructions. The position of the sphere achieving the highest/lowest RC is depicted by a separate colored boxes for each sphere. For reconstructions without resolution modeling (Fig. 6a), it can be observed that the position of the sphere achieving the highest/lowest RC is the same for all spheres. The highest RC occurred for spheres positioned at the top of the phantom, while the lowest RCs occurred for spheres positioned laterally at the bottom. By application of resolution modeling (OSEM_*RR*) also a fixed position for the highest/lowest RCs can be observed for spheres with a diameter larger than 13 mm. The lowest RCs occurred for spheres positioned at the top of the phantom, while the highest RCs occurred for spheres positioned centrally at the bottom. It is also noticeable in these reconstructions that the volume of the two directly adjacent spheres appears to have an influence on RC. There is a tendency for the smallest RC to occur when the two largest remaining spheres are direct neighbors. In contrast, the highest RC occurs when the two smallest remaining spheres are direct neighbors.

### Influence of the sphere positioning on the fit of the RC curve

The mean, standard deviation, maximum and minimum of the fit parameters for all 720 permutations are given in Table 2. It can be seen that the sphere positioning also has a non-negligible influence on the fit parameters, which is reflected in a standard deviation and variation of the fit parameters. The variation of $\beta$ and $\gamma$ (${\vartheta }_{\beta }$ and ${\vartheta }_{\gamma }$, see Eq. 5) is considerably larger when resolution modeling is applied. No systematic difference in the variation of both parameters is observed between both phantoms.

**Table 2:** Statistical analysis of the fitting process

Fitting parameter	Phantom type	Reconstruction	Mean	Standard deviation	Minimum	Maximum	Variation
$\beta$	NEMA PET	OSEM_*noRR*	2.89	0.07	2.74	3.09	12.1%
		OSEM_*RR*	1.08	0.07	0.82	1.21	36.1%
	NEMA SPECT	OSEM_*noRR*	3.07	0.04	2.97	3.18	6.8%
		OSEM_*RR*	1.09	0.12	0.66	1.39	67.0%
$\gamma$	NEMA PET	OSEM_*noRR*	1.98	0.14	1.65	2.33	34.3%
		OSEM_*RR*	2.45	0.38	1.51	4.00	101.6%
	NEMA SPECT	OSEM_*noRR*	1.76	0.12	1.44	2.02	33.0%
		OSEM_*RR*	2.14	0.37	1.31	3.64	109.9%

Mean, standard deviation, maximum/minimum and variation (see Eq. 5) of the fitting parameters $\beta$ and $\gamma$ used to fit ${f}_{RC}\left(d\right)$ for all 720 permutations are given for both phantoms (NEMA PET, NEMA SPECT) and both reconstructions (OSEM_*noRR*, OSEM_*RR*)

**Fig. 7:**
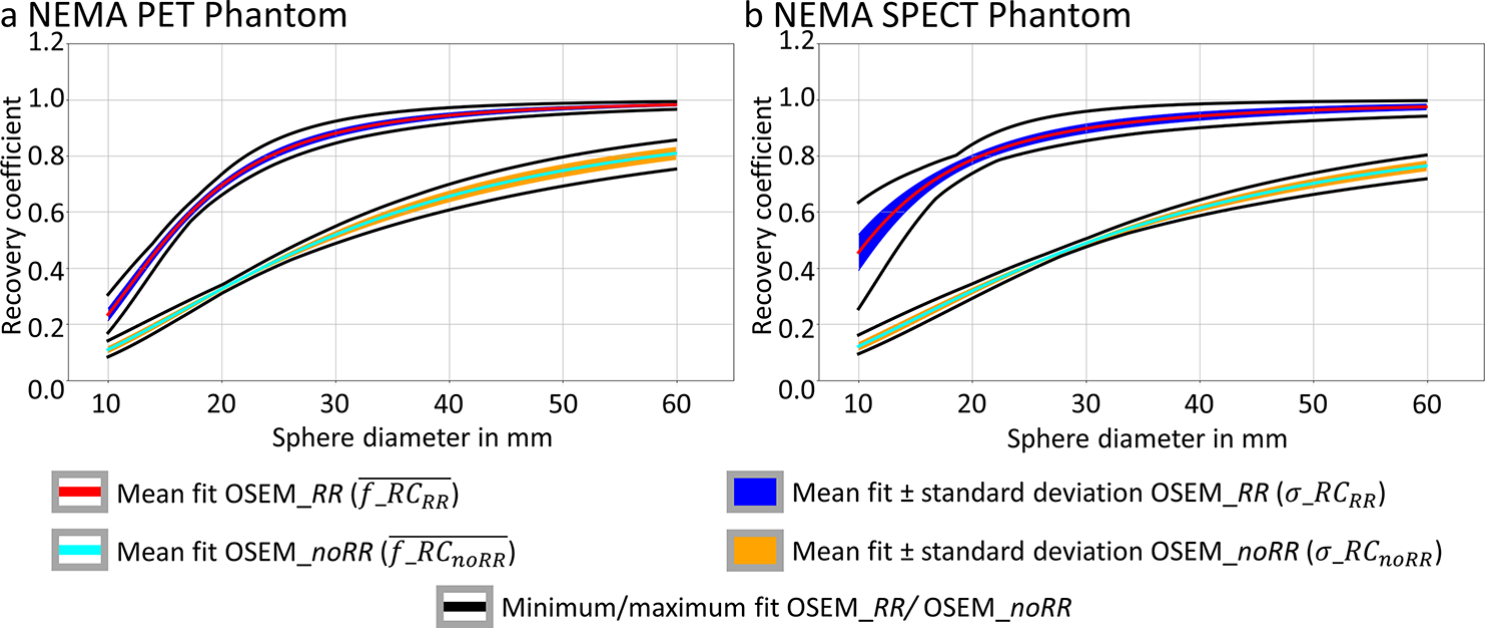
Mean, standard deviation and maximum/minimum RCs of the fits for the NEMA PET Phantom (**a**) and the NEMA SPECT Phantom (**b**). Cyan/red lines represent the means of $\overline{{f}_{RC}}\left(d\right)$ for OSEM_*noRR*/OSEM_*RR* reconstructions. Orange/blue areas illustrate the range of one standard deviation ($\overline{{f}_{RC}}\left(d\right)$ ± ${\sigma }_{RC}\left(d\right)$). The maximum/minimum ${f}_{{RC}_{i}}\left(d\right)$ values for both reconstructions are illustrated by black lines

Figure 7 shows the mean fit $\overline{{f}_{RC}}\left(d\right)$ of the RC curve for both phantoms and both reconstruction types. In addition, the range of one standard deviation ($\overline{{f}_{RC}}\left(d\right)$ ± ${\sigma }_{RC}\left(d\right)$) and the maximum and minimum ${f}_{{RC}_{i}}\left(d\right)$ values are illustrated. Again, it can be observed that different sphere positioning leads to a range of different fits of the RC curves. For OSEM_*noRR* (orange), this range appears wider for larger sphere diameters and narrower for smaller diameters. For OSEM_*RR*, the opposite case can be observed.

The ${r}^{2}$ values for OSEM_*noRR* (NEMA PET: 0.96, NEMA SPECT: 0.98) were significantly higher (paired Wilcoxon test, *p* < 0.001) than for OSEM_*RR* (NEMA PET: 0.92, NEMA SPECT: 0.90). In addition, for OSEM_*noRR*, the ${r}^{2}$ value for the NEMA SPECT Phantom was significantly larger than for the NEMA PET Phantom. In contrast, the opposite was observed for OSEM_*RR*.

### Analysis of ^177^Lu SPECT/CT measurements for different sphere configurations

**Fig. 8:**
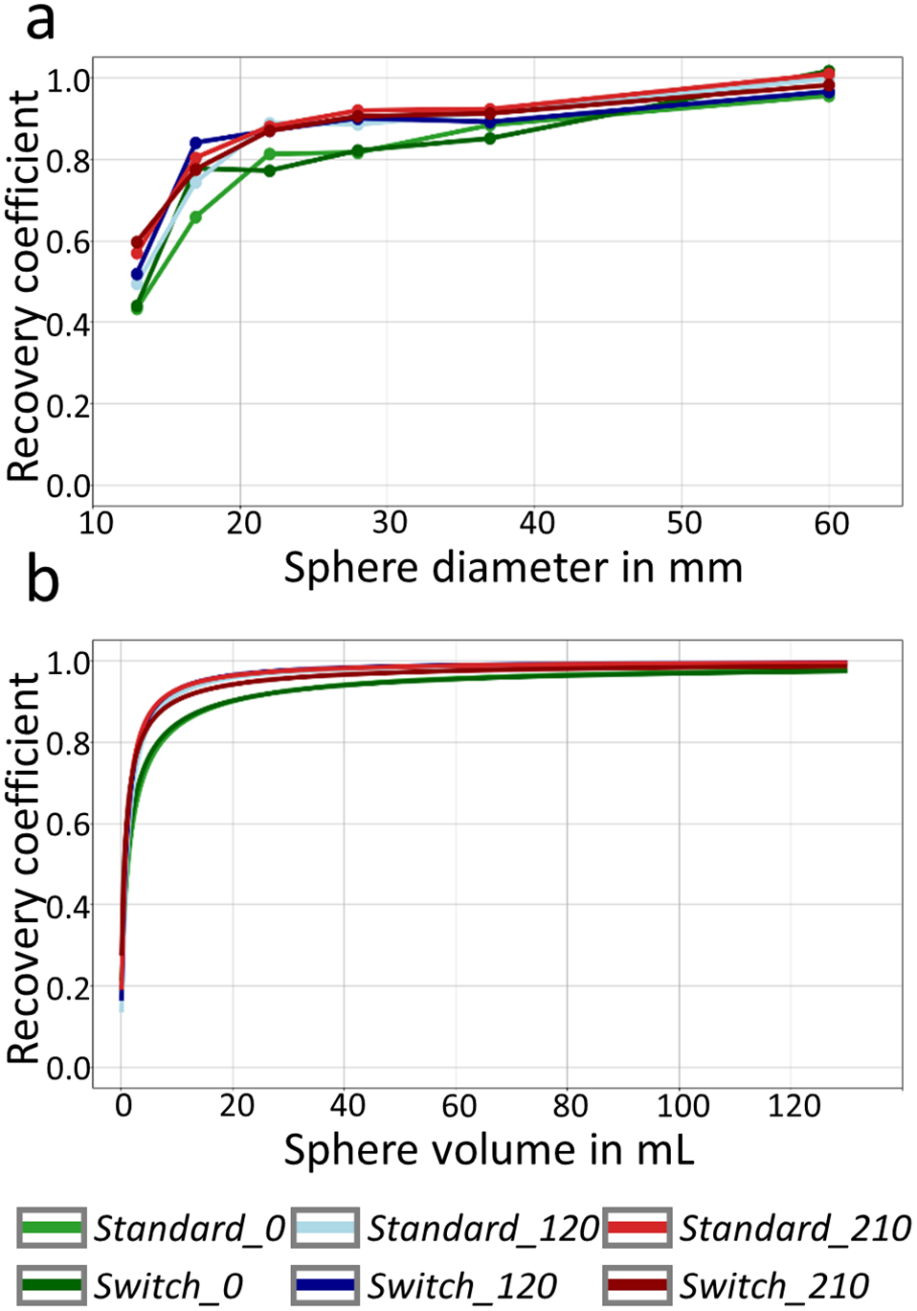
RC curves (**a**) and fit of the corresponding RC curves (**b**) for all six measured sphere configurations. **a**: RCs of the sphere configurations presented in Fig. 3. **b**: fitted RC curves for different configurations, obtained by performing a fit of the RC curve using the fit function given by Eq. 4. The parameter of the fits are given in Table 3

Figure 8 shows the RCs of the six different sphere configurations (see Fig. 3). As for the simulations, the sphere positioning has a decisive influence on RC. This also affects the fits of the RC curves, as can be seen from the different fit curves in Fig. 8b) on the one hand, and the different values of the fit parameters $\beta$ and $\gamma$ in Table 3 on the other hand. Thus, the influence of sphere positioning on the RCs observed in the simulations is confirmed by the ^177^Lu SPECT/CT measurements.

**Table 3:** Fitting parameters for the measured RC curves

Configuration	$\varvec{\beta }$	$\varvec{\gamma }$	*r* ^2^
Standard	1.37	2.44	0.96
Switch	1.27	2.27	0.85
Standard 120	1.27	3.21	0.96
Switch 120	1.20	3.18	0.85
Standard 210	1.15	3.04	0.96
Switch 210	1.07	2.42	0.99

Fit parameters β and γ and coefficient of determination ${r}^{2}$ of the fitted RC curves (Fig. 8b) for all six sphere configurations

The values for ${\updelta }\left(d\right)$ (Eq. 6), ${\Delta }\left(d\right)$ (Eq. 7) and $R{C}_{mean}\left(d\right)$ are given in Table 4 for all six spheres. It can be seen that ${\updelta }\left(d\right)$ is smaller than ${\Delta }\left(d\right)$ for all spheres. This means that the deviations between measurements and simulation are considerable smaller than the mean deviation between all performed measurements. The deviation of the simulation therefore has a smaller effect on the spread of recovery coefficients compared to the effect of different sphere arrangements.

**Table 4:** Quantitative parameters to determine accuracy of simulations

Sphere diameter d in mm	${\updelta }\left(d\right)$	${\Delta }\left(d\right)$	*RC*_mean_(*d*)
13	0.039	0.053	0.51
17	0.021	0.044	0.77
22	0.019	0.038	0.85
28	0.010	0.037	0.87
37	0.019	0.021	0.90
60	0.018	0.020	0.99

Quantitative parameters ${\updelta }\left(\text{d}\right)$, ${\Delta }\left(\text{d}\right)$ and $R{C}_{mean}\left(d\right)$ for each sphere to determine the accuracy of the simulations performed

## Discussion

We demonstrated that the positioning of spheres in the NEMA phantom considerably influences the expected recovery. This applies to both types of phantoms (NEMA PET Phantom and NEMA SPECT Phantom with a large 60 mm sphere replacing the standard 10 mm sphere) and regardless of whether resolution modeling is applied. The variation in RC is considerably larger for smaller spheres than for larger spheres. When comparing the two reconstruction types, several observations were made. For the reconstructions without RR, maximum or minimum RCs were obtained when the sphere was positioned at the top or laterally at the bottom, respectively. These are the positions where the distance between the sphere’s center and the detector is at its minimum or maximum, respectively (see depiction of trajectory at the bottom of Fig. 6). This outcome was expected because the resolution of SPECT projections depends on the distance to the detector and OSEM_*noRR* reconstruction does not account for this distance-dependent resolution. Therefore the best spatial resolution is expected at the top of the phantom, as for this position the partial volume effect is smallest, resulting in the largest RC. The exact opposite is true for the position at the lateral bottom. Here, the detector has the largest distance to the sphere center. The worst spatial resolution is to be expected here, which in turn results in the lowest RC. In contrast, the reconstruction with RR (OSEM_*RR*) models the PSF of the imaging system with a function dependent on the distance to the detector. Therefore, the resolution should have a lower dependence on the distance to the detector. Still, it was observed that maximum/minimum values were obtained for certain positions of the sphere. However, this could not be explained by the distance of the detector to the surface. Additionally, for the OSEM_*RR* reconstruction, it was observed that the volume of neighboring spheres does influence the RCs (see Fig. 6). On one hand, this could be due to the convergence of OSEM_*RR*. For positions where large spheres are direct neighbors, more iterations are needed for convergence. The underlying reason is the multiplicative nature of the updating process leading to the reconstruction method being non-linear. If a large number of updates is used, the effects of non-linearity are suppressed. Modeling spatial resolution in the backprojector slows convergence [29], and hence a much larger number of updates needs to be employed for all objects to converge. In our analysis, we chose a relatively high number of 200 updates, which is rather uncommon in clinical practice (for example 6i6s = 36 updates are used clinically at University Hospital Würzburg and 16i5s = 80 updates at University Hospital Leuven, respectively). Reconstructions with higher update numbers would require significantly more time and amplify Gibbs artifacts. This will therefore increase noise in the SPECT images, which can be regularized by performing post-filtering [30]. Another reason for the dependence of the RCs on the size of the neighboring spheres could be the non-uniform convergence of the OSEM algorithm. A large object can superimpose the counts for a small object in the projections and therefore decrease the convergence rate of this smaller object (see Supplemental Material Figure S3). This hypothesis is supported by the fact that for the NEMA PET Phantom the mean RCs are larger than for the NEMA SPECT Phantom RCs for the same sphere size (see Table 1). Our analysis also demonstrates that the decreasing effect of a directly neighboring sphere on recovery is considerably stronger than the increasing effect of spill-in. If the latter had a greater impact, one would have observed an increase in RC for large neighboring spheres. Since the opposite was observed in our analysis, the distance between the spheres in the NEMA phantom is presumably still sufficiently large (8.5 mm distance between the surfaces when the two largest spheres are direct neighbors; approximately 2 cm distance between the surfaces when the smallest sphere is neighboring the largest sphere) that the increasing effect of spill-in is too small to cancel out the decreasing effect of the large sphere.

**Fig. 9:**
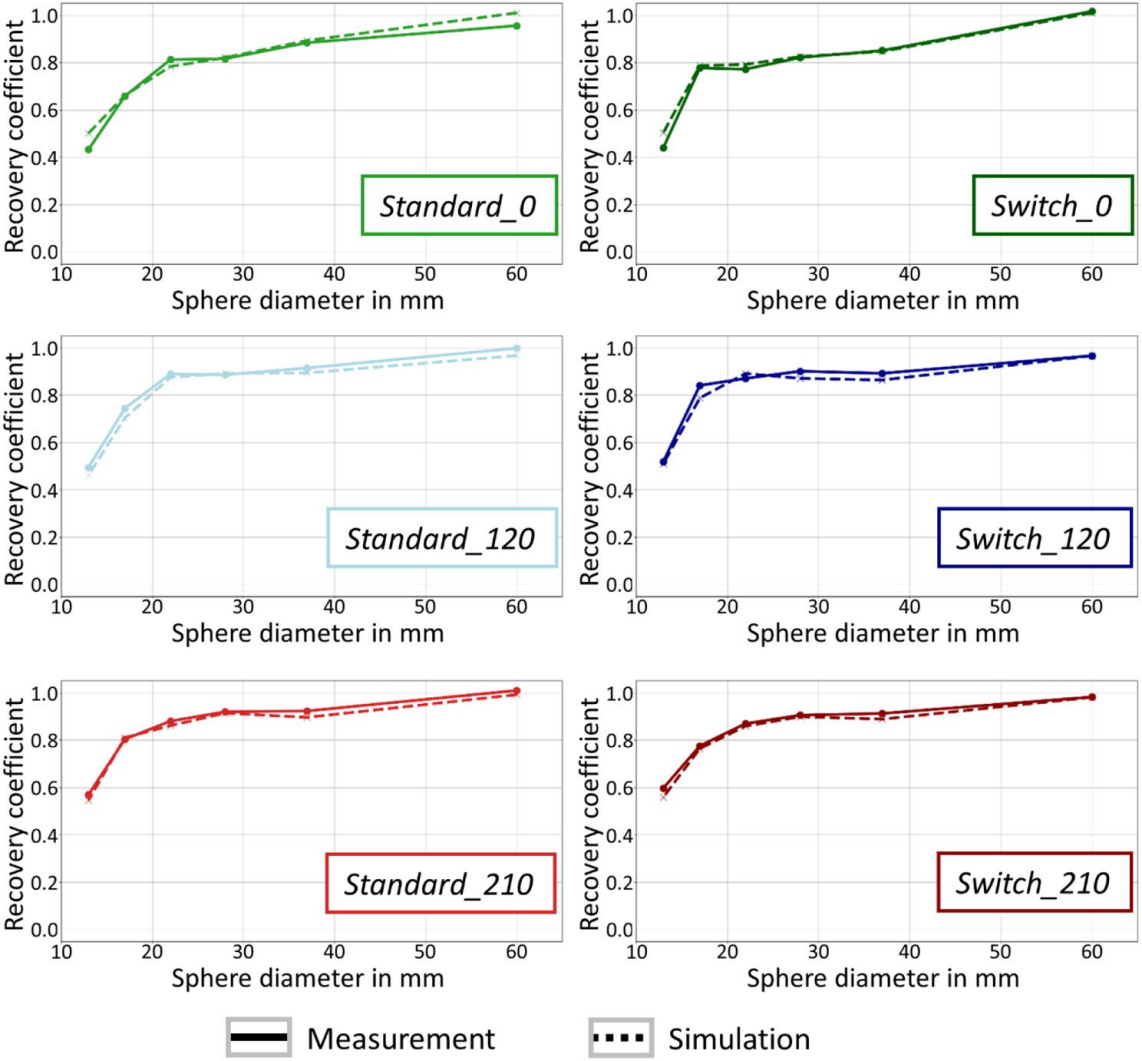
Comparison between measured and simulated RC curves for six different sphere positioning. Each plot shows the RC curve of the measured (solid line) and the simulated (dashed line) SPECT acquisitions. In the top row the plots for *Standard*, *Rotation 1* and *Rotation 2* (from left to right) are given. In the bottom row, the switched versions are given (*Switch Standard*, *Switch Rotation 1*, and *Switch Rotation 2*). The measured RC curves were calculated on Flash3D reconstructions with 160 updates, the simulated RC curves on STIR reconstructions with 160 updates

Based on ^177^Lu SPECT/CT measurements of six different sphere configurations, it was demonstrated that the dependence of RCs on the sphere positioning, one of the key findings of the simulations, can also be observed in physical phantom measurements. Although the simulations closely followed the general shape of the measured RC curves (see Fig. 9), there were some differences between simulation and measurement. The possible sources of error can be divided in three categories:

1. Intrinsic limitations: In our analysis we compared two different reconstruction programs (STIR and Flash3D), which have shown very similar, but not exactly equal results. Also it is almost impossible to model a SPECT/CT system perfectly by MC simulations. There will be always sources of errors like collimator and crystal imperfections, sensitivity differences and the behavior of the photomultiplier tube.
2. Deviations between simulations and SPECT measurements: There are differences between the used parameters for the simulations (adjusted to the SPECT system at University Hospital Würzburg) and the real conditions for the measurements performed at the University Hospital Leuven such as slightly different detector orbits or detector energy resolutions. Both systems should perform in a similar manner, as the same SPECT model is used, but there are still some deviations which might be eliminated by fine-tuning the simulations. Furthermore, noise added to the simulations and inherent to the physical-phantom measurements causes small differences in measured recoveries. This effect is likely small for the large spheres, but could have a larger effect for the smaller spheres, due to the higher susceptibility of the small volume to outliers. The disparity in voxel dimensions between the simulation (4.8 mm) and measurement (2.4 mm) may also be a contributing factor. The smaller voxel size results in a lower number of counts per voxel, thereby reducing the signal-to-noise ratio. Consequently, in practice, the larger voxel size is typically employed.
3. Uncertainties during the measurement: Registration errors, attenuation map errors, different positioning of the phantom and air bubbles can lead to uncertainties, which will in turn lead to slightly different results every time a measurement is repeated.

The findings in this study have direct implications for a future accreditation program aimed at harmonizing quantitative SPECT/CT imaging. Due to the variations in RC, a fixed sphere configuration would need to be defined, non-compliance with which (i.e., sites measuring the SPECT NEMA Phantom with a different sphere configuration) would result in the measurement having to be repeated with the specified configuration. However, experience with PET/CT accreditation has shown that misunderstandings in positioning can very easily occur and otherwise cleanly performed accreditation measurements would then have to be rejected. In order to avoid such problems and thus increase the acceptance of accreditation, a number of possibilities would exist to slightly soften such a strict accreditation: One possibility could be to simply expand the range of accepted RCs based on the RC variation observed in the simulations. Another option would be to leave the sphere positioning open to the sites and then transform the RC curve for the chosen configuration to a predefined standard configuration. The good agreement between simulation and measurement (see Fig. 9) indicates that such a transformation might be feasible. However, practical implementation is non-trivial and would require further investigation in subsequent studies. The accreditation of a site would then be based on the RC curves transformed to the standard configuration. In conclusion, our study demonstrates that to obtain dosimetry results that can be compared between centers, standardization and harmonization should not be limited to image acquisition, but also needs to include image processing.

In addition to the influence on the RCs, it has been shown that the sphere positioning also affects the fit of the RC curves. This, in turn, affects a potential partial volume correction of SPECT images using RC-PVC. The implications will be explained through a brief example calculation: Site A and Site B have conducted ^177^Lu SPECT/CT measurements of the NEMA SPECT Phantom to perform partial volume correction using the fit of the RC curve. All relevant measurement parameters are identical between sites A and B, except for the sphere configuration inside the NEMA Phantom. Site A uses the *Standard* sphere configuration, while Site B uses the *Standard_120* configuration (see Fig. 3 for both configurations). Both sites perform RC-PVC for a spherical lesion with a diameter of 2.67 cm (corresponds to a sphere volume of 10 mL). Using the fit parameters from Table 3 and the fit function in Eq. 4, the theoretical RC for this sphere can be calculated. RCs of 0.84 and 0.92 are calculated and for Site A and B, respectively. This results in a correction by a factor of 1.19 for Site A and 1.09 for Site B. Consequently, partial volume correction would result in a 9.2% higher total activity in the lesion for Site A with respect to Site B. This difference in quantitative activity determination results solely from the use of different sphere configurations in the NEMA SPECT Phantom measurement. Furthermore, a phantom measurement does not necessarily fully capture all other physical effects that may deteriorate SPECT-based activity estimation, e.g., scatter. Whilst typically not considered a PVE, insufficient scatter management with window-based methods may compromise the transferability of results between geometries [31]. While effective scatter source estimation (ESSE) has been successfully employed for ^177^Lu SPECT/CT imaging [32], the majority of clinical sites utilize triple-energy window scatter estimation. Therefore, we elected to utilize the most clinically utilized scatter method in our study. Therefore, our analysis raises questions about the reliability of the application of RCs from previous phantom measurements for PVC in SPECT imaging especially for small volumes. Consequently, performing PVC based on a single phantom measurement can lead to substantial dose errors for small lesions. For more accurate dose calculations, PVC should take into account tumor location and surrounding activity, which would require a wide range of phantom measurements or accurate RC modeling. A further option is to account for the background activity by modifying the RC to a contrast recovery coefficient [21], which is consistent with the experimental findings of Staanum [33]. In practice, it might be necessary to modify the linear relationship [21] between background-to-object ratio and RC [34].

## Conclusion

Based on ^177^Lu SPECT/CT simulations of different sphere configurations in a NEMA Phantom, this study shows that the sphere positioning has a significant influence on the recovery coefficients. These findings were validated by ^177^Lu SPECT/CT measurements of six different sphere configurations. Based on these results, sphere positioning should definitely be considered in a possible future accreditation procedure for quantitative ^177^Lu SPECT/CT to allow harmonization between different sites as well as image processing. It was also shown that the variation of recovery coefficients also has a large influence on the fit of the recovery curves. In consequence, RC-PVC, when used in ^177^Lu SPECT/CT imaging, crucially depends on the arrangement of spheres in the underlying NEMA Phantom measurement. Furthermore, the single-measurement method normally performed for PVC should be reconsidered to account for the position dependency.

### Electronic supplementary material

Below is the link to the electronic supplementary material.

Supplementary Material 1

## Data Availability

The datasets used and/or analyzed during the current study are available from the corresponding author on reasonable request.

## Abbreviations

MRT: Molecular radiotherapy
EARL: EANM Forschungs GmbH
NEMA: National Electrical Manufacturers Association
IEC: International Electrotechnical Commission
RC: Recovery coefficient
PVE: Partial volume effect
PSF: Point spread function
PVC: Partial volume correction
VOI: Volume of interest
RR: Resolution recovery
MC: Monte Carlo
OSEM: Ordered-subset expectation maximization
AC: Attenuation correction
SC: Scatter correction
ESSE: Effective scatter source estimation
