# Supplementary material for: Position dependence of recovery coefficients in 177Lu-SPECT/CT reconstructions – phantom simulations and measurements
Source: EJNMMI Phys. 2024 Jun 28;11:52. doi: 10.1186/s40658-024-00662-y (PMC11211301; doi:10.1186/s40658-024-00662-y)
Supplement: Supplementary file 1 — Supplementary Material 1 [file 40658_2024_662_MOESM1_ESM.pdf]

Position dependence of recovery coefficients in ^{177}Lu-SPECT/CT reconstructions – phantom simulations and measurements -Supplemental Material-

Julian Leube[1] Wies Claeys[2] Johan Gustafsson[3] Michael Lassmann[1]
Maikol Salas-Ramirez[1] Michel Koole[2] Johannes Tran-Gia[1]

[1] Department of Nuclear Medicine, University Hospital Würzburg, Würzburg, Germany

[2] Department of Imaging and Pathology, KU Leuven, Leuven, Belgium

[3] Medical Radiation Physics Lund, Lund University, Lund, Sweden

In order to reduce the size of the main manuscript, it was decided to include parts of the methods and results in the supplemental material.

1 Methods

1.1 Determination of the center of the spheres

The sphere centers were determined based on the CT using a semi-automatic segmentation method written in Python. A threshold of 30 HU was used to identify the sphere walls (all voxels with HU

values greater or equal to 30 were assigned a value of 1, and all remaining voxels were assigned a value of 0). Possible holes in the wall masks were manually adjusted by setting these values from 0 to 1. Next, the spheres were automatically filled out and the center of each sphere was calculated as the center of mass (R_j) of each sphere mask ($j = 1, \ldots, 6$):

$$\boldsymbol{R_j} = \frac{\sum_{i=1}^{N} m_i \boldsymbol{r_i}}{\sum_{i=1}^{N} m_i} \tag{1}$$

Here, $m_i \in \{0, 1\}$ is the value of the ith voxel in the coarse segmentation mask and $\boldsymbol{r_i}$ is the position of the ith voxel. After determining the center $\boldsymbol{R_j}$ for all six spheres, a fine segmentation mask M_j was created for each sphere j by setting all pixels i to 1, which fulfill the following equation:

$$(\boldsymbol{R_j} - \boldsymbol{r_i})^2 \leq \left(\frac{d_j}{2}\right)^2 \tag{2}$$

Here, d_j is the diameter of sphere $j = 1, \ldots, 6$. Voxels that did not satisfy this equation were set to 0. The fine segmentation mask M_j has the same resolution and matrix size as CT-HD. Based on M_j, the recovery coefficient (RC_j) was calculated for each sphere j using the performed SPECT/CT reconstructions. Before calculating the RCs, all SPECT reconstructions were interpolated to CT-HD resolution using tri-linear interpolation. For the SPECT/CT measurements of the six sphere configurations,performed in Leuven, SPECT reconstructions were performed using the vendor reconstruction Flash3D. These SPECT images were additionally registered to the CT using tools available in MIM. The RC_j of each sphere j was then calculated by dividing the SPECT-based activity (fine segmentation mask, M_j multiplied voxelwise with the SPECT reconstruction, A) by the nominal activity within the sphere, $A_{\text{True},j}$:

$$RC_j = \frac{\sum_{i=1}^{K} M_{j,i} A_i}{A_{\text{True},j}} \tag{3}$$

$A_{\text{True},j}$ was calculated by multiplying the sphere volume V_j with the activity concentration of the SPECT/CT acquisition.

2 Results

2.1 Influence of the number of OSEM updates on the recovery coefficients

In the main manuscript, only the convergence of the mean RC across all spherical permutations was analysed. In addition, an analysis on the variation of the RC changes as a function of the number of updates of the OSEM reconstruction is performed. Figure S1 shows the variation of the RC for each sphere of the NEMA SPECT Phantom from 20 to 200 updates of the OSEM_RR reconstruction as a boxplot.

Especially for the small spheres (diameter $< 28\,\mathrm{mm}$), it can be seen that the size of the boxes becomes smaller with increasing number of updates. Increasing the number of updates therefore reduces the influence of sphere positioning on the variation of the RC. On the other hand, for the large spheres (diameter $\geq 28\,\mathrm{mm}$), no change in the variation of the RC can be observed after a certain number of updates. It can therefore be assumed that the observed dependence of the RC on the positioning of the spheres is also present for a large number of updates of the OSEM_RR reconstruction.

2.2 Variation of recovery coefficient for different sphere positioning

For reasons of space, not all relevant statistical values of the RC for all permutations are given in the main manuscript. Instead, the full version of Table 1 can be found in the Supplemental Material Table S1.

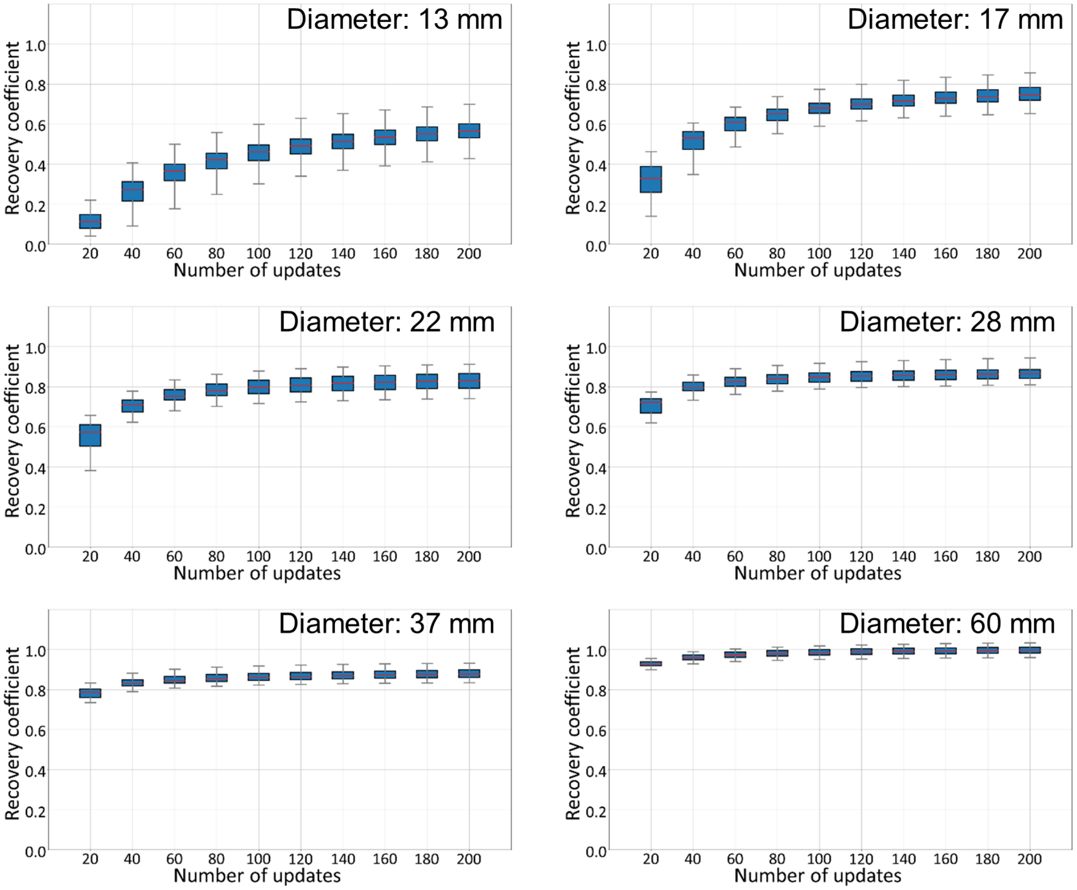

Figure S1: Boxplots for each sphere diameter showing the RCs of all permutations plotted against the number of updates of the OSEM_RR reconstruction (phantom type: NEMA SPECT Phantom). The red lines represent the median RC of all 720 permutations. The blue boxes represent the interquartile range of the RCs. The whiskers correspond to the maximum/minimum values for each number of updates.

Phantom type	Reco type	Sphere diameter	Mean ($\overline{RC}$)	Standard deviation (σ_{RC})	Minimum (RC_{min})	Maximum (RC_{max})	RC variation (θ_{RC})
NEMA PET	OSEM_*noRR*	10	0.090	0.017	0.047	0.133	96 %
		13	0.161	0.022	0.117	0.216	61 %
		17	0.267	0.027	0.216	0.323	40 %
		22	0.388	0.028	0.338	0.444	27 %
		28	0.501	0.027	0.450	0.546	19 %
		37	0.596	0.025	0.547	0.635	15 %
	OSEM_*RR*	10	0.410	0.046	0.287	0.535	60 %
		13	0.642	0.052	0.516	0.797	44 %
		17	0.787	0.051	0.678	0.893	27 %
		22	0.846	0.045	0.752	0.940	22 %
		28	0.879	0.033	0.811	0.956	16 %
		37	0.892	0.025	0.843	0.942	11 %
NEMA SPECT	OSEM_*noRR*	13	0.098	0.017	0.050	0.158	110 %
		17	0.183	0.017	0.137	0.239	56 %
		22	0.292	0.017	0.252	0.344	32 %
		28	0.406	0.017	0.371	0.455	21 %
		37	0.517	0.017	0.484	0.559	15 %
		60	0.718	0.016	0.685	0.754	10 %
	OSEM_*RR*	13	0.566	0.050	0.427	0.699	48 %
		17	0.751	0.043	0.653	0.856	27 %
		22	0.829	0.042	0.742	0.913	21 %
		28	0.868	0.032	0.810	0.944	15 %
		37	0.880	0.025	0.835	0.933	11 %
		60	0.997	0.025	0.961	1.034	7 %

Table S1: Statistical analysis of the RCs for all 720 permutations. The mean RC ($\overline{RC}$), its standard deviation (σ_{RC}), the maximum/minimum RC (RC_{min}/RC_{max}), and the RC variation (θ_{RC}) of all 720 permutations are given for the two different phantom types (NEMA PET, NEMA SPECT) and both reconstructions (OSEM_*noRR*, OSEM_*RR*).

2.3 Influence of the sphere positioning on the fit of the RC-volume curve

Fits for the RC curve of each sphere permutation were performed as described in the main manuscript. The mean, standard deviation and maximum/minimum values of the fit parameters were then determined. Another possibility of performing the fit is to fit the mean RC ($\overline{RC}$) considering the standard deviation of the RC (σ_{RC}) as the error. The fits for both phantom (NEMA PET and NEMA SPECT) and reconstruction types (OSEM_*noRR* and OSEM_*RR*) are shown in Figure S2. The parameters of the fits are given in Table S2.

Table S2: Fit parameters β and γ ($\pm$ uncertainities) and coefficient of determination r^2 of the fitted RC curves (Fig. S2) for both phantom and reconstruction types.

Phantom type	Reconstruction	β	γ	r^2
NEMA PET	OSEM_*noRR*	2.02 ± 0.13	2.89 ± 0.09	0.99
	OSEM_*RR*	1.07 ± 0.07	2.07 ± 0.25	0.94
NEMA SPECT	OSEM_*noRR*	3.09 ± 0.10	1.76 ± 0.12	0.99
	OSEM_*RR*	1.07 ± 0.11	1.98 ± 0.28	0.95

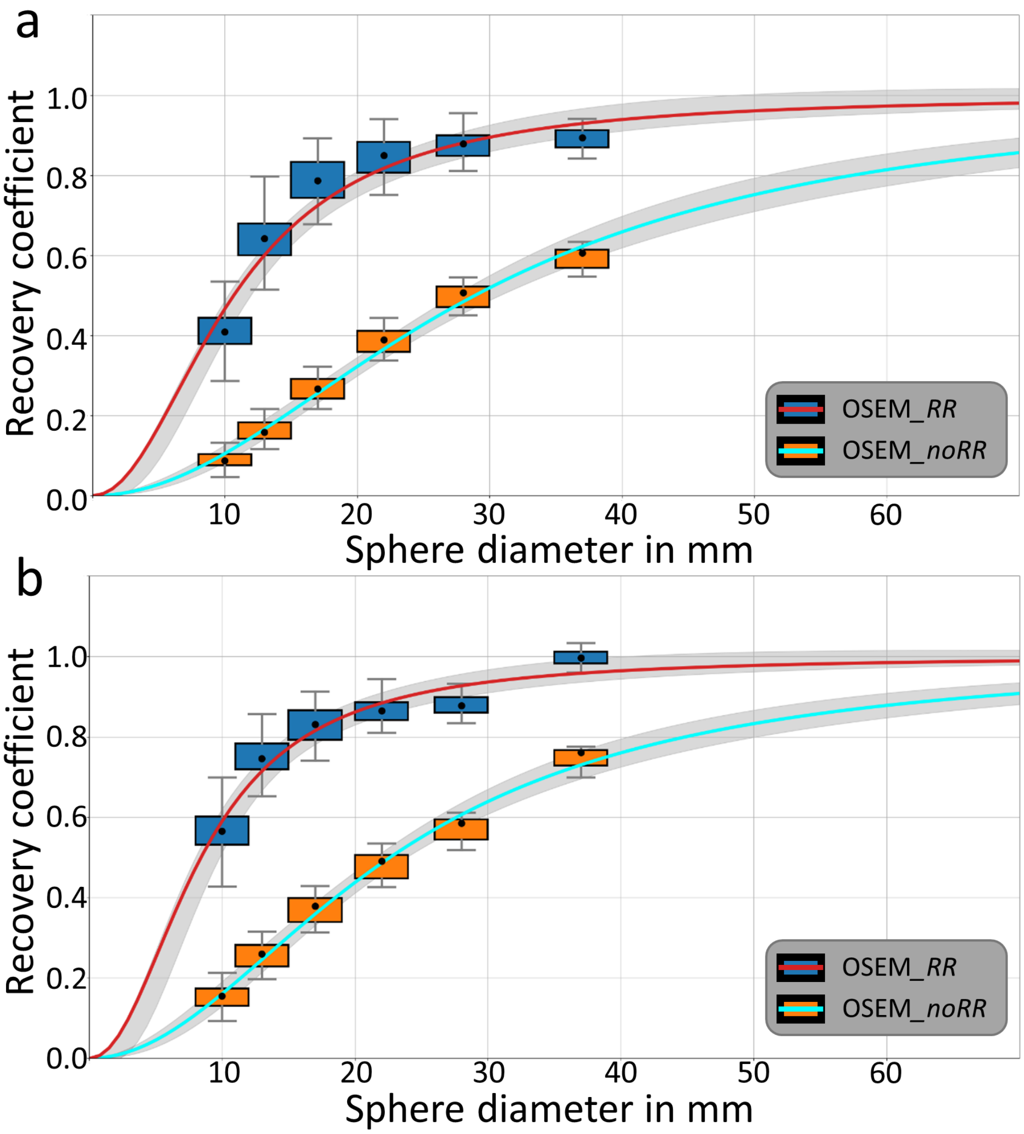

Figure S2: Boxplots and fit of the mean RC for each sphere diameter showing the RCs of all permutations of the OSEM_*noRR* and OSEM_*RR* reconstruction (a: NEMA PET, b: NEMA SPECT). The black dots represent the median RC of all 720 permutations. The blue/orange boxes represent the interquartile range of the RCs. The whiskers correspond to the maximum/minimum values for each sphere diameter. The cyan/red line represent the fit of the mean RC curves regarding the standard deviation as an error. The gray area corresponds to the 95% confidence interval.

3 Simulation to determine the effect of large hot objects on the convergence of small objects

To assess the influence of neighbouring spheres on the convergence of the OSEM reconstruction for small spheres, additional Monte Carlo simulations were performed. The arrangement of the simulations is shown in Figure S3 a. For Simulation A, a 13 mm diameter sphere was placed in the centre of the NEMA phantom. Two large spheres of 60 mm diameter were placed above and below the small sphere. The large spheres were placed on the same ring on which the spheres of the NEMA SPECT phantom are typically located. For Simulation B, the small sphere was simulated without any neighbouring activity. The simulations were performed using SIMIND with the same parameters as for the main simulations of the study. Subsequently, SPECT reconstructions were performed with RR (STIR, 1 subset, iterations from 20 to 1000, step size of 20 iterations) and without RR (CASToR, 1 subset, iterations from 10 to 100, step size of 10 iterations). Finally, the RC of the small sphere was calculated for both simulation arrangements (A/B), both reconstruction types and all update numbers.

The RCs as a function of the number of updates are shown in Figure S3 b (left: OSEM_$noRR$; right: OSEM_RR). For both reconstruction types, Simulation A results in lower RCs. For OSEM_$noRR$, convergence is observed after approximately 20 updates for Simulation B, while Simulation A has not fully converged even after 100 updates. For OSEM_RR, the convergence of Simulation B is achieved after about 400 updates. Simulation A achieves a lower RC after 1000 updates than Simulation B after only 20 updates.

Furthermore, it can be observed that OSEM_$noRR$ results in significantly lower recovery values for both simulation setups compared to OSEM_RR. The achieved values are even lower than those in the NEMA phantom. This can be explained by the positioning of the small sphere for this simulation. In the center of the NEMA phantom, the worst spatial resolution is expected, due to the large distance between the sphere and the collimator. For OSEM_RR this is not an issue, as this effect is corrected by the resolution modeling. Nevertheless, the ideal RC of 1 could not be obtained for this simulation. This can be caused by inaccuracies in the PSF modeling correction (PSF is

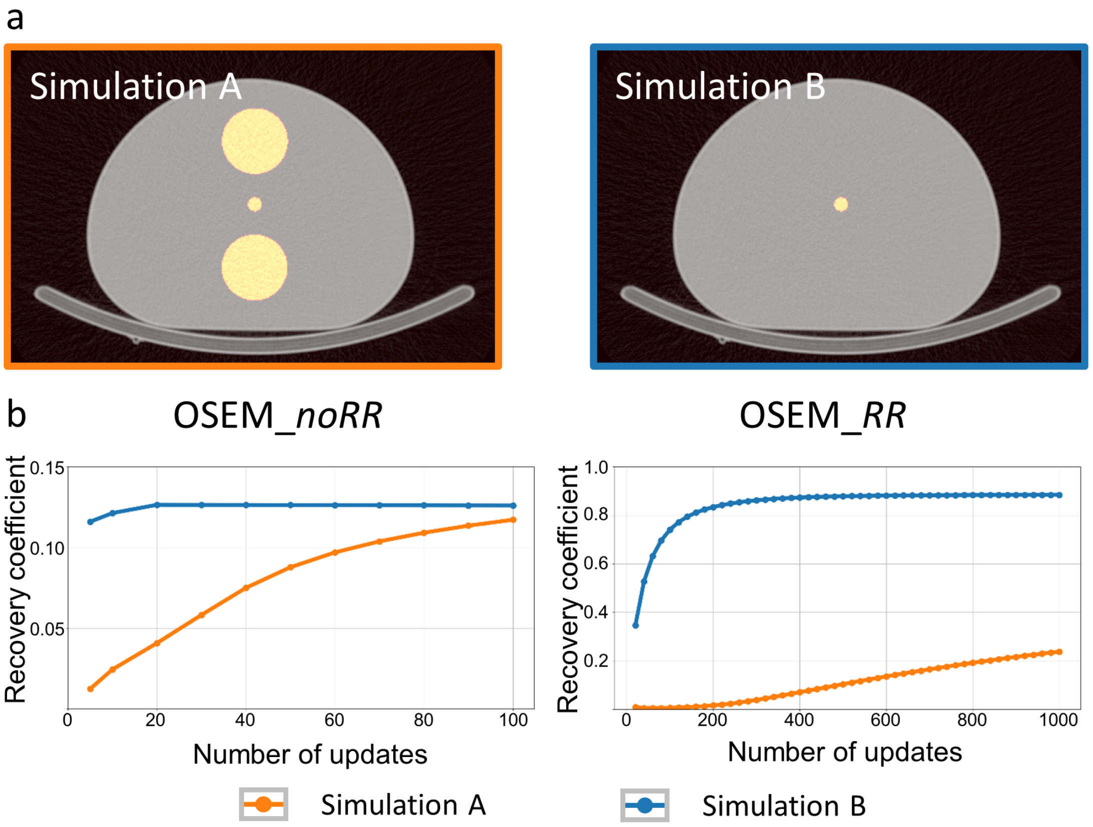

Figure S3: Simulation to determine the effect of large hot objects on the convergence of small objects. a: overview of the two arangements used to perform the simulations. b: RC of the small sphere in the center as function of the number of updates for both simulation arrangements (Simulation A: orange curve; Simulation B: blue curve) and both reconstruction types (left: OSEM_*noRR*, right: OSEM_*RR*)

modeled as a Gaussian function in STIR, with its FWHM linearly increasing with the distance of the detector), inaccuracies in determining the mask (in SIMIND, the spheres are geometrically defined and are therefore ideal, but the mask for determining the recovery is based on the SPECT voxels, resulting in a sampling effect), or inaccuracies in scatter correction.

The simulations impressively demonstrates that large hot objects strongly influence the convergence of small objects in OSEM reconstructions. This is true for both reconstructions without and with RR.
